# Alternative use of *Bacillus subtilis* spores: protection against environmental oxidative stress in human normal keratinocytes

**DOI:** 10.1038/s41598-018-20153-2

**Published:** 2018-01-29

**Authors:** Ganna Petruk, Giuliana Donadio, Mariamichela Lanzilli, Rachele Isticato, Daria Maria Monti

**Affiliations:** 1 Department of Chemical Sciences, University of Naples Federico II, Complesso Universitario Monte Sant’Angelo, via Cinthia 4, 80126 Naples, Italy; 2 Department of Biology, University of Naples Federico II, Complesso Universitario Monte Sant’Angelo, via Cinthia 4, 80126 Naples, Italy; 3 Istituto Nazionale di Biostrutture e Biosistemi (INBB), Rome, Italy

## Abstract

Inorganic trivalent arsenic is a major environmental pollutant and exposure to human results in many pathologies, including keratosis and carcinoma. Here, we analyzed the effects of *B. subtilis* spores on human normal keratinocytes in the presence of sodium arsenite oxidative stress. Pre-treatment of cells with spores before inducing oxidative stress was able to keep normal levels of intracellular ROS, GSH and lipid peroxidation, as well as to inhibit the activation of the MAPK cascade. Moreover, spores showed a positive effect on cell proliferation, probably due to their binding on the cell surface and the activation of intracellular catalases. We found that spores exert their protective effect by the nuclear translocation of Nrf-2, involved in the activation of stress response genes. This, in turn, resulted in a protective effect against sodium arsenite stress injury, as oxidative stress markers were reported to physiological levels when cells were stressed before incubating them with spores. Therefore, *B. subtilis* spores can be considered as a new agent to counteract oxidative stress on normal human keratinocytes.

## Introduction

Arsenic is a natural element widely present in food, water, air and soil^1^. The inorganic form exists predominantly in trivalent (As^3+^, such as sodium arsenite and arsenic trioxide) or pentavalent (As^5+^) form^2^ and is generally considered more harmful than organic forms^3^. Epidemiological studies have shown that chronic exposure to trivalent arsenite is associated with dermal toxicity, neurodegenerative disorders, cardiovascular diseases and the increased incidence of cancer in lung, skin, bladder, and liver^4–6^. The major form of trivalent arsenite is sodium arsenite (herein denoted as SA), a water contaminant, known to induce several human diseases^5,7^. Indeed, SA carcinogenicity has been evaluated by International Agency for Research on Cancer (IARC) for the first time in 1973^3^. Moreover, it has been reported that the exposure of human cell lines to SA increases the production of reactive oxygen species (ROS)^8–10^, which induce intracellular oxidative stress and result in oxidative DNA damage and end into apoptosis^11,12^.

Cells are equipped with an array of antioxidant systems, as the superoxide dismutases (SODs), which catalyze the dismutation of superoxide anions into H_2_O_2_ and oxygen, maintaining a low intracellular ROS level. H_2_O_2_ is reduced by various systems, mainly by catalases and peroxidases. However, these endogenous systems are often insufficient for complete scavenging of ROS. Thus, endogenous or exogenous antioxidants have been proposed to be potentially beneficial in reducing SA-induced toxicity. Nowadays, a continuous search for new products able to prevent or retard stress-induced damages is still needed. Here, we propose the use of bacterial spores of *Bacillus subtilis* to counteract SA-induced damage.

Bacteria spores are metabolically dormant. They are produced by members of various *Bacillus* species in response to harsh environments^13^. The spore can survive in this state for long time, resisting to a vast range of stresses, such as high temperature, dehydration, absence of nutrients and presence of toxic chemicals^14^. When the environmental conditions ameliorate, spores germinate, thus giving rise to vegetative cells able to grow and to sporulate again in case conditions should require it.

Some *Bacillus* species are on the Food and Drug Administration’s GRAS (generally regarded as safe) list and several spore-based products are widely commercialized as probiotics for human and animal use^15,16^. It has been shown that the ingestion of spores produced by *B. subtilis*, the model organism for spore formers, restores the normal microbial flora following extensive antibiotic use or illness^17^. *B. subtilis* is a ubiquitous bacterium found on skin, in the digestive tract, in epithelial wounds, on extremities of the human body, in livestock and in soil^18,19^. Thus, it has developed adaptive strategies to subsist in different environments *via* the production and secretion of a large number of molecules that could exert a probiotic activity in the host^20,21^. In this article, the bacterial spores are used in a different field, i.e. that of exploiting their formidable resistance properties to prevent sodium arsenite oxidative stress in epithelial cells. Human keratinocytes have been selected as model epithelial cells since these cells are normally present in the outermost layer of the skin and more exposed to environmental stress.

## Results

### Effects of *B. subtilis* spores on human normal keratinocytes

**Figure 1:**
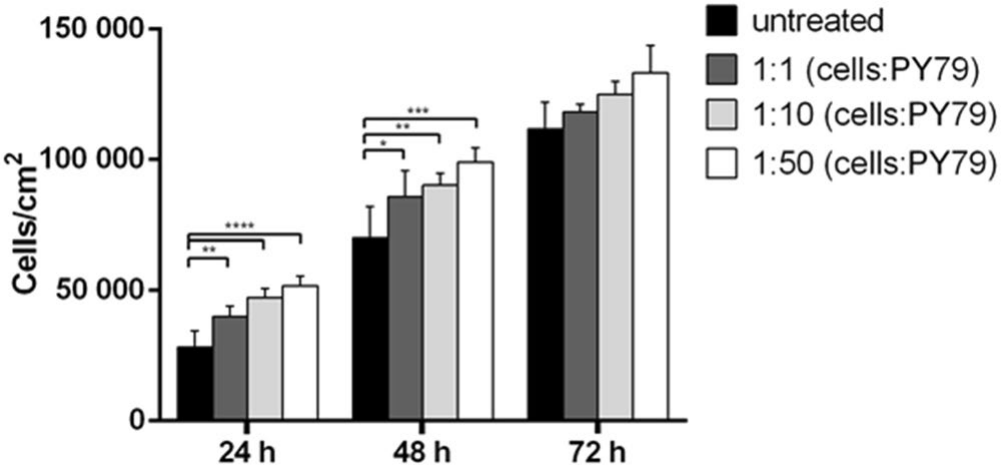
Effects of PY79 spores on human normal keratinocytes. Dose-response bar blot of HaCaT cells after 24, 48 and 72 h incubation in the presence of different concentrations of PY79 spores, in a ration from 1:1 to 1:50 (cells:spores). Cell survival percentage was defined as reported in Methods section. Values are given as means ± S.D. (n ≥ 3). *Indicates p < 0.05; **Indicates p < 0.005, ***Indicates p < 0.001, ****Indicates p < 0.0001, with respect to untreated cells of each time point.

Human keratinocytes (HaCaT cells) were chosen because normally exposed to different environmental stress, so they can be considered as guard cells of the body. The biocompatibility of wild type spores of *B. subtilis* (PY79) on HaCaT cells was tested by a time-course and dose-response test, using a ratio from 1:1 to 1:50 (cells:spore). As shown in Fig. 1, spores had a positive effect on cell viability, as an increase of about 60% and 30% was observed after 24 and 48 h, respectively, at the highest ratio used. No significant increase in cell viability was observed after 72 h, as normal cells are sensitive to the contact inhibition phenomenon, stopping their growth when they become confluent. On the basis of these results, subsequent experiments were carried out at a ratio 1:50 (cells:spore).

It has been reported that *B. subtilis* spores are not able to properly germinate and outgrowth in cell culture medium for at least 5 hours^22^. In our experimental conditions, a partial loss of spore-refraction was observed after 24 h incubation, in the presence of 5% CO_2_, in complete medium, in the absence or presence of HaCaT cells. After 72 h incubation, the number of black spores represented the 90% of the total spores and no vegetative cells were observed. These data suggest that spores are able to start the germination process, but not the outgrowth, according to previously published data^22^.

### Pretreatment with *B. subtilis* spores inhibits SA-induced damage in eukaryotic cells

**Figure 2:**
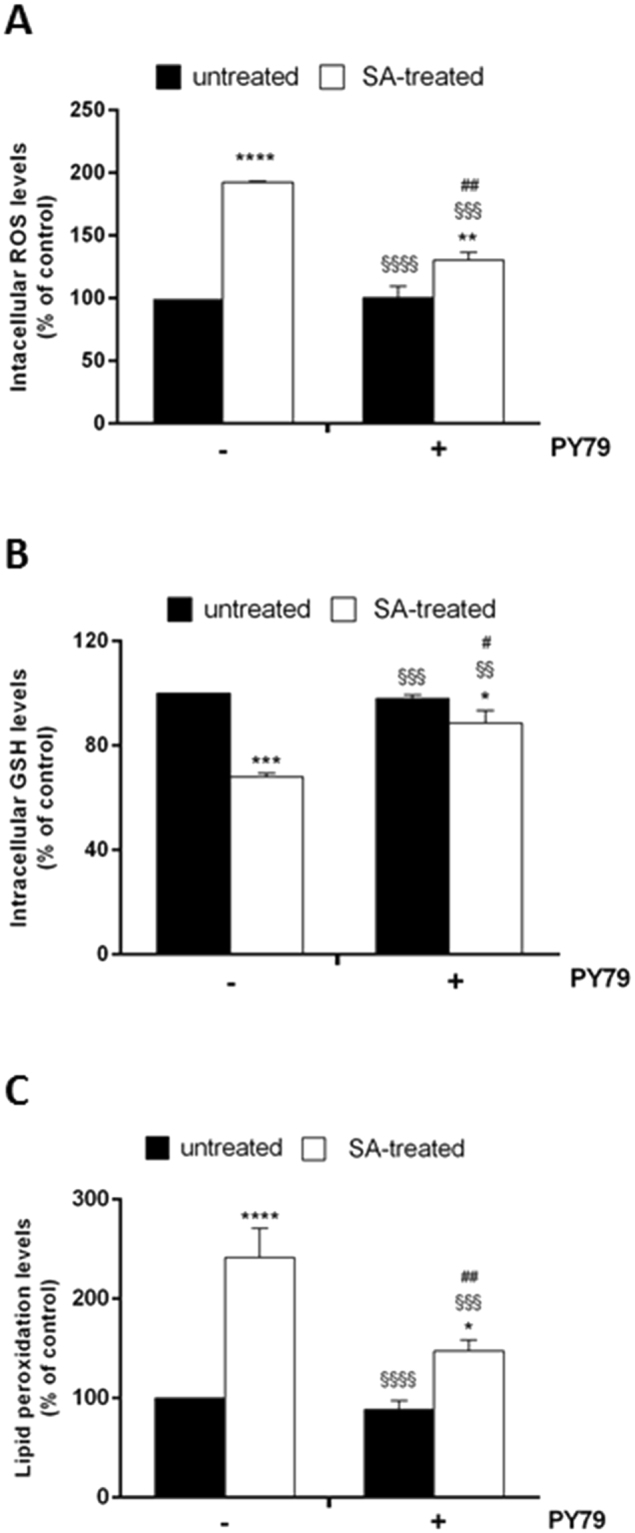
Analysis of the oxidative stress markers in HaCaT cells exposed to SA treatment in the presence of PY79 spores. Cells were pre-incubated in the presence of PY79 (1:50, cells:spores) for 30 min and then 300 µM SA was added to the culture medium for 45 min. (**A**) intracellular ROS levels were determined by DCFDA assay; (**B**) intracellular GSH levels determined by DTNB assay; (**C**) lipid peroxidation levels determined by TBARS assay. Values are expressed as fold increase with respect to control cells. Data shown are the means ± S.D. of three independent experiments. *Indicates p < 0.05, **Indicates p < 0.005, ****Indicates p < 0.0001, with respect to control cells; ^§§^Indicates p < 0.005, ^§§§^Indicates p < 0.001, ^§§§§^Indicates p < 0.0001, with respect to SA-treated cells; ^#^Indicates p < 0.05; ^##^Indicates p < 0.005 with respect to PY79-treated cells.

Trivalent inorganic arsenic (iAs^3+^) is a toxic and carcinogenic environmental contaminant that humans are inadvertently exposed to every day through water, food and air. Epidemiological investigations demonstrate that long-term exposure to SA leads not only to different types of cancer in skin, but also in lung, liver, kidney and bladder^5^. SA exerts its toxic effect through ROS generation that, produced in the mitochondria, cause loss of GSH homeostasis and oxidations of molecules (such as formation or lipid peroxides)^23^. To analyze the effects of *Bacillus* spores on cells subjected to oxidative stress, HaCaT cells were pretreated with PY79 spores for 30 min and then stressed by using 300 µM SA. Immediately after oxidative stress induction, ROS production was determined by using H_2_DCFDA (2,7-dichlorofluorescin diacetate) (Fig. 2A). As expected, 45 min treatment with SA induced a 90% increase in ROS intracellular levels compared to control cells (Fig. 2A). Interestingly, when cells were incubated with spores before SA treatment, no increase in ROS level was observed (p < 0.001). This finding suggests that mitochondrial dysfunction, induced by SA treatment, may be prevented by PY79 through a ROS-mediated signaling pathway. It is known that GSH is the most abundant low molecular weight thiol that plays important roles in redox, nutrient metabolism, and regulation of cellular events^24^, and is oxidized during oxidative stress. Thus, we analyzed intracellular GSH content in cells incubated in the presence of spores. Following SA-oxidative stress induction, we found a 30% decrease (p < 0.001) in intracellular GSH levels with respect to control cells, whereas GSH levels were unaltered in cells incubated with spores and then stressed (Fig. 2B). The anti-stress activity of spores was further confirmed by TBARS assay, in which the peroxidation level of lipids was analyzed. The result of the experiment is reported in Fig. 2C and it clearly shows that administration of spores was able to keep unaltered lipid peroxidation levels. In fact, cells pre-treated with spores and then exposed to SA stress showed significantly lower intracellular levels of lipid peroxidation (100%, p < 0.01) if compared to untreated cells exposed to SA.

**Figure 3:**
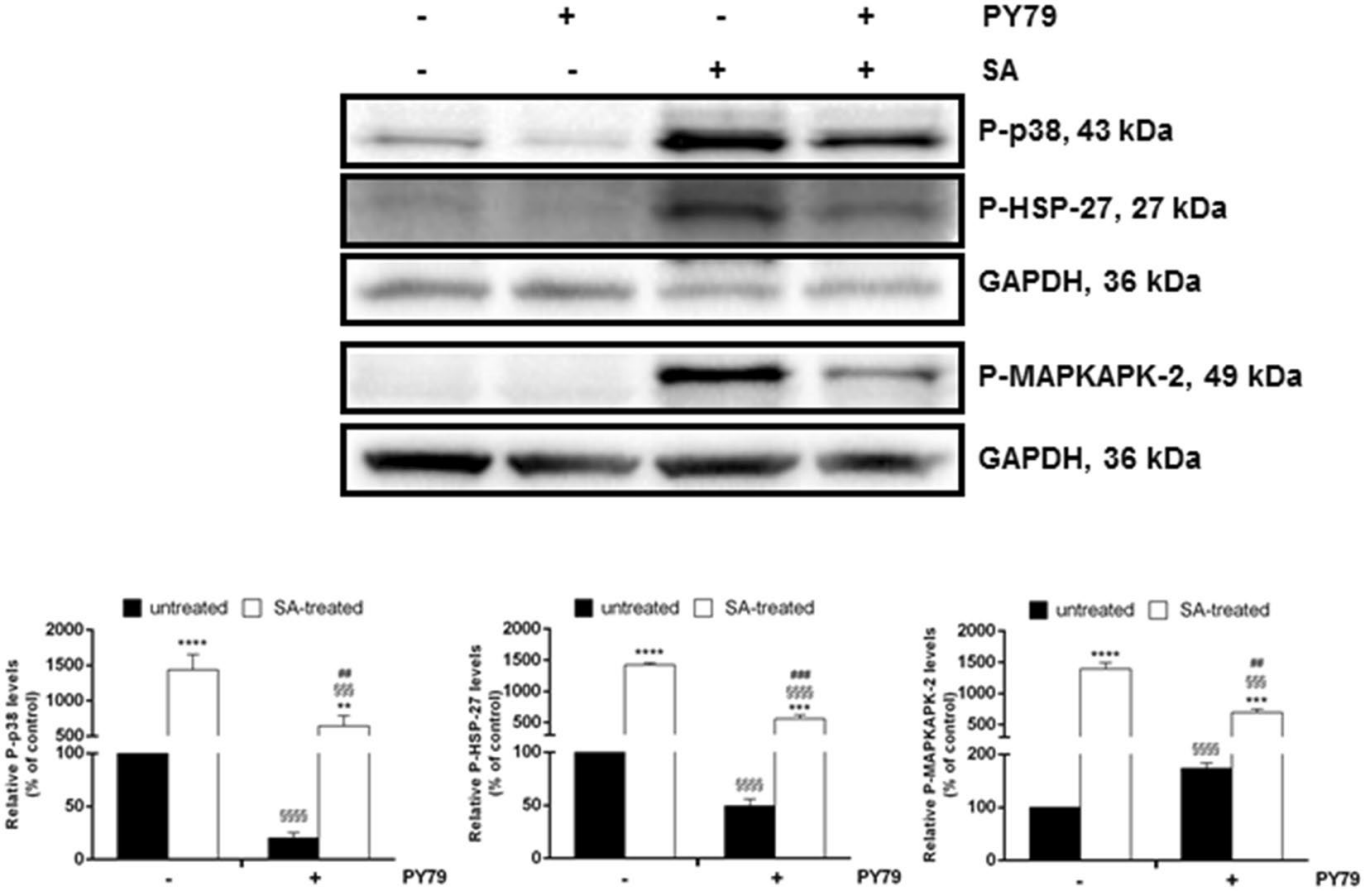
Effect of spores on SA-induced oxidative stress markers in HaCaT cells. Western blots show the phosphorylation levels of p38 (upper panel), HSP-27–2 (middle panel) and MAPKAPK (lower panel), with the relative densitometric analysis in the absence (black bars) or in the presence (white bars) of SA. GAPDH was used as internal standard. Full-length blots are presented in Supplementary Figure S2. **Indicates p < 0.005, ***Indicates p < 0.001, ****Indicates p < 0.0001, with respect to control cells; ^§§§^Indicates p < 0.001, ^§§§§^Indicates p < 0.0001, with respect to SA-treated cells; ^##^Indicates p < 0.005, ^###^Indicates p < 0.001, with respect to PY79-treated cells.

The protective effect of *B. subtilis* spores was finally confirmed by Western blot experiments. In particular, we analyzed the phosphorylation levels of p38, its direct target, MAPKAPK-2 and HSP-27 (Fig. 3). These proteins are important members of the mitogen-activated protein kinase (MAPK) family, and are directly involved in oxidative signaling stress pathways^25^. Under stress conditions, we observed a significant increase in the phosphorylation levels of these three proteins, as expected (Fig. 3, third lanes). On the other hand, co-treatment of cells with PY79 spores and SA resulted in the inhibition of the phosphorylation of the analyzed markers (Fig. 3, fourth lanes). No oxidative stress was observed when cells were incubated with spores. Even though we cannot exclude that the levels of the proteins studied, rather than their phosphorylated forms, might vary, we believe that the alteration in the phosphorylation level of the three markers is due to SA treatment, as SA is known to induce phosphorylation of p38 and its targets^26,27^. Therefore, the present study suggests that skin toxicity induced by exposure to arsenic can be easily counteracted by adding *B. subtilis* spores. This new use of spores would be a novel strategy to protect derma cells from SA toxicity.

To verify whether the observed protective effect of the spores was not specific for the cell line or the induced stress type, we repeated the experiments using a different cell line, i.e. human colon cancer (LoVo) cells, stressed by SA, a well-known water contaminant^5^. Alternatively, HaCaT cells were exposed to a different oxidative stress inducer, i.e. UVA radiation, which normally reaches the outermost layer of the skin^25^.

HaCaT cells were incubated with increasing number of spores (from ratio 1:1 to 1:50) for 30 min and then stressed by UVA irradiation (20 J/cm^2^). After 90 min, cell lysates were analyzed by Western blotting. UVA induced a significant increase in the phosphorylation level of p38, whereas incubation of cells with spores prior to UVA irradiation, resulted in a dose-dependent decrease of p38 phosphorylation level. In particular, in the ratio 1:50, this level was similar to that observed in non-irradiated cells (Fig. S1A, p < 0.0001).

As for LoVo cells, cells were co-treated with 300 µM SA and spores and then intracellular ROS, GSH levels and lipid peroxidation were analyzed. As shown in Fig. S1(B–D), no alteration of these markers was observed during co-exposure of cells to SA spores, suggesting a general efficacy of spores in counteracting oxidative stress (Fig. S1).

### *B. subtilis* spores are able to heal keratinocytes after SA injury

**Figure 4:**
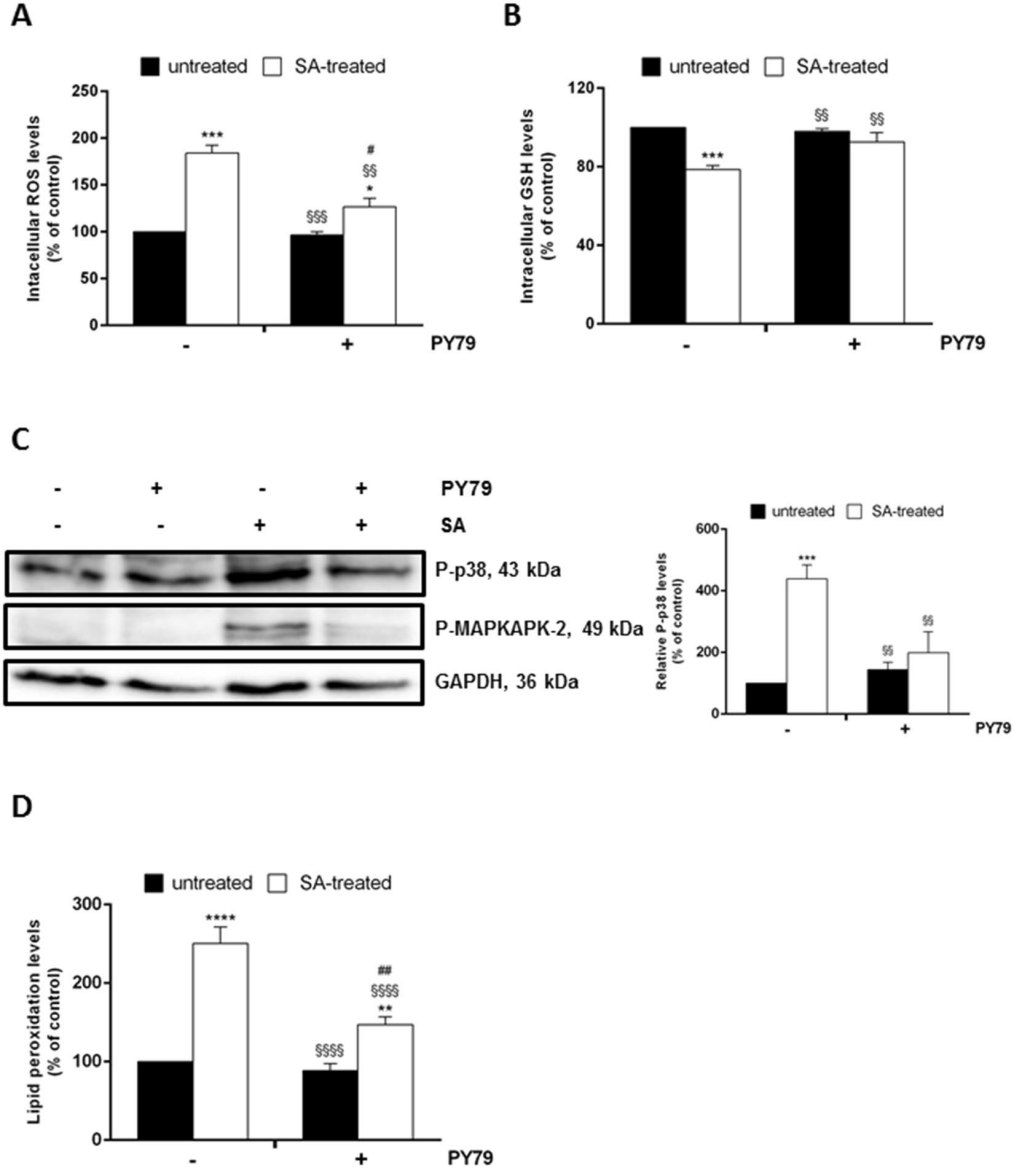
Ability of spores to heal SA-induced oxidative stress in HaCaT cells exposed to SA treatment. Cells were stressed by 300 µM SA for 45 min and then incubated in the presence of PY79 (1:50, cells:spores) for 30 min. (**A**) intracellular ROS levels were determined by DCFDA assay; (**B**) intracellular GSH levels determined by DTNB assay; (**C**) Western blots show the phosphorylation levels of p38 (upper panel), MAPKAPK-2 (middle panel), with the relative densitometric analysis in the absence (black bars) or in the presence (white bars) of SA. GAPDH was used as internal standard. Full-length blots are presented in Supplementary Figure S2; (**D**) lipid peroxidation levels determined by TBARS assay. Values are expressed as fold increase with respect to control cells. Data shown are the means ± S.D. of three independent experiments. *Indicates p < 0.05, **Indicates p < 0.01, ***Indicates p < 0.001, ****Indicates p < 0.0001, with respect to control cells; ^§§^Indicates p < 0.005, ^§§§^Indicates p < 0.001, ^§§§§^Indicates p < 0.0001, with respect to SA-treated cells; ^#^Indicates p < 0.05, ^##^Indicates p < 0.005, with respect to PY79-treated cells.

In the search of a general anti-stress application of spores, we evaluated if PY79 spores could restore cells after SA injury. To this purpose, cells were first stressed by SA for 45 min and then incubated for 30 min with spores. ROS production, GSH oxidation, activation of MAPK cascade and lipid peroxidation were evaluated at the end of incubation (Fig. 4). Interestingly, SA-induced alteration in ROS and GSH levels was suppressed by the presence of spores, as ROS and GSH levels were similar to those obtained for untreated and for spore-treated cells (Fig. 4A,B). Accordingly, a decrease in the phosphorylation level of p38 and in lipid peroxidation levels was detected after SA injury and spore-treatment (Fig. 4C,D).

### The spore-induced antioxidant activity is regulated by Nrf-2 nuclear translocation

**Figure 5:**
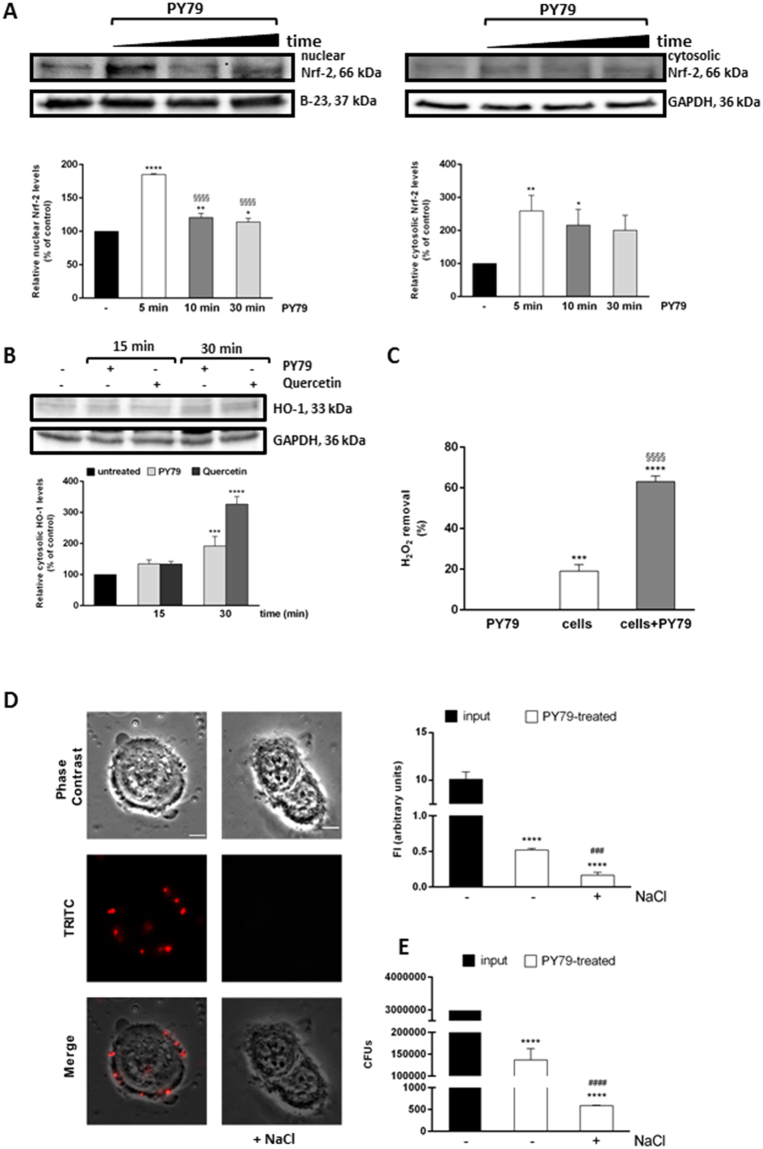
PY79 spores effects on HaCaT cells. (**A**) Western blot analysis for Nrf-2 was performed on cytosolic and nuclear proteins obtained from HaCaT cells after incubation with spores (ratio 1:50) for 5 min (white bars), 15 min (dark grey bars) and 30 min (light grey bars). Nrf-2 was quantified by densitometric analysis and normalized to GAPDH (cytosol) or B-23 (nucleus). (**B**) Western blot analysis for HO-1 was performed on total proteins obtained from HaCaT cells after incubation with spores (ratio 1:50, light grey bars) or quercetin (25 µM, dark grey bars) for 15 min and 30 min. HO-1 was quantified by densitometric analysis and normalized to GAPDH. Full-length blots are presented in Supplementary Figure S2. Data shown are the means ± S.D. of three independent experiments. *Indicates p < 0.05, **Indicates p < 0.005, ***Indicates p < 0.001 ****Indicates p < 0.0001, with respect to control cells; ^§§§§^Indicates p < 0.0001, with respect to PY79-treated cells for 5 min. (**C**) Catalase assay on HaCaT cells in the presence of PY79 spores. Cells were exposed to spores (1:50) for 30 min and then 50 µg of cell lysates were incubated with 0.036% (w/w) H_2_O_2_. Then, H_2_O_2_ concentration in solution was determined by measuring the absorbance at 240 nm. The experiments were performed in triplicate and the data were expressed as the mean of three independent experiments. ***Indicates p < 0.001, ****Indicates p < 0.0001, with respect to spores; ^§§§§^Indicates p < 0.0001, with respect to control cells. (**D**), Adhesion of spores to HaCaT cells. Cells were incubated with PY79 fluorescent spores (1:50, cells:spores) for 30 min, treated in the presence or absence of 0.6 M NaCl in PBS, washed, fixed and observed by fluorescence microscopy, as described in Methods section. A representative image is reported for both the experimental conditions tested. The same field has been observed by phase contrast and fluorescence microscopy. Merged panels are reported. The exposure time was of 200 ms. Scale bar 1 mm. Histograms on the right represent quantitative data obtained by spectrofluorimetric analysis. Values are expressed as fluorescence intensity. Data shown are the means ± S.D. of three independent experiments. (**E**) Determination of the number of spores bound to HaCaT cells. Input, total number of PY79 spores used. In (**D** and **E**), ****Indicates p < 0.0001, with respect to control cells; ^###^Indicates p < 0.001, ^####^Indicates p < 0.0001 with respect to PY79-treated cells without NaCl treatment.

In order to get insights into the molecular mechanism of *B. subtilis* spores-protective effect, we analyzed the involvement of the transcription factor Nrf-2. Under normal physiological conditions, Nrf-2 is associated to Keap-1, which keeps Nrf-2 in the cytosol and directs it to proteasomal degradation. Upon either oxidative stress induction and/or in the presence of antioxidants, Keap-1 dissociates from Nrf-2. Nrf-2 is thus translocated to the nucleus, binds to antioxidant responsive element (ARE) sequences and activates the transcription of several antioxidant enzymes^28^. It has been recently reported that lactobacilli, well-known probiotics, can elicit their beneficial effect, in intestinal epithelium cells, upon NOX binding^29,30^. This binding induces the generation of near threshold levels of ROS, sufficient to induce Nrf-2 nuclear translocation^29,30^. Therefore, we incubated HaCaT cells in the presence of PY79 spores for different length of time (from 5 min to 30 min) and lysates were analyzed by Western blotting using a Nrf-2 antibody. As shown in Fig. 5A, an increase in both cytosolic and nuclear Nrf-2 levels was observed after 5 min incubation, and then the nuclear signal decreased over time. Nrf-2 activation was confirmed by measuring heme oxygenase 1 (HO-1) level. This protein was chosen as it has been reported that the Nrf-2/ARE mediate the expression of many detoxification enzymes, such as HO-1 and SOD^31^. We found that a significant increase in HO-1 level was observed after 30 min incubation of cells in the presence of spores (Fig. 5B). In order to confirm the specificity of HO-1 activation, 25 µM quercetin was used as a positive control. This antioxidant molecule is known to activate Nrf-2 pathway and to induce an increase in HO-1 level^32,33^. Nrf-2 activation was further confirmed by measuring the H_2_O_2_ consumption, directly related to the catalase activity. As reported in Fig. 5C, the catalase activity detected in keratinocyte lysates after incubation with spores was higher than in untreated cells (p < 0.0001). One should consider that no catalase activity was detected in *B. subtilis* spores when 1 × 10^9^ spores were tested (Fig. 5C). Moreover, the antioxidant activity observed is not related to spores’ intracellular enzymes, as spores are resistant to the lysis procedure followed for eukaryotic cells^34^. Spores ability to bind specifically to the cells was analyzed by using spores bound to fluorescent proteins^35^. Cells were incubated as described above, in the presence or absence of 0.6 M NaCl. High salt treatment was used to remove specifically bound spores, as this is a well-established procedure to remove surface bound ligands^36^. The results shown in Fig. 5D indicated that about 5% of the total amount of fluorescently labeled spores were specifically bound to the cell surface, and that, after incubation with NaCl, a significant decrease in fluorescence intensity was observed (p < 0.001).

Finally, the number of spores bound to the surface of HaCaT cells was measured by performing another experiment. Cells were treated as described above, but at the end of incubation, cells were detached by trypsin. The pellet, containing cells and the spore bound to the cells, was plated on LB-agar plates. Then, colonies were counted and the total colony forming units (CFUs) were reported in the histograms of Fig. 5E. Results clearly indicated that about 5% of initial spores remained bound to the cells, and after NaCl treatment, a significant decrease in CFUs was observed. These results were perfectly in agreement with those obtained in Fig. 5D.

## Conclusions

Trivalent arsenite is a severe environmental injury to which humans are normally exposed. It has been associated with many disorders, from cardiovascular to cancer^4–6^. Human population is mostly exposed to SA through inhalation, ingestion and dermal contact^37^. Several lines of evidence demonstrated that exposure of human cell lines to SA induces an increase in ROS levels, DNA damage and leads to apoptosis^8–12^. In this context, we investigated the possibility of an alternative and innovative use of *B. subtilis* spores. In particular, using an array of biochemical methodologies, we analyzed the protective effect of spores in counteracting the SA-induced oxidative stress on human normal keratinocytes. Spores clearly induced a positive effect both in the prevention of stress as well as when used to heal cells after stress injury. This applicability was found to be independent of the cell type used and the source of stress, as the same protective effect was observed when colon cells were stressed by SA and when UVA was used on HaCaT cells (see Fig. S1). It is worth to notice that the protective effect exerted by spores is not only due to the antioxidant activity of the spore, but to the activation of Nrf-2, a nuclear transcription factor, actively involved in oxidative stress response^28^. As already reported, gut bacteria (as *B. subtilis* and *L. plantarum*) stimulate ROS production in epithelial cells by an enzymatic mechanism analogous to the pathogen-induced respiratory burst in phagocytes^29,38^. Interestingly, enzymatically generated ROS in the epithelia are stimulated not only by potential pathogens, but also by symbiotic bacteria, especially members of the *Lactobacilli* taxon. Bacteria promote cell proliferation and migration^39,40^, accelerate restitution post injury^41^ and modify epithelial NF-kB signaling^42^. Recently, Jones and co-workers demonstrated that the presence of *L. plantarum* was able to slightly increase ROS production, just enough to promote the dissociation between Keap-1 and Nrf-2^30^. The increase in ROS levels is mediated by the catalytic action of NADPH oxidases, such as Nox-1, present on the cell membrane. The expression of Nox enzymes has been found in barrier cells, including phagocytes, colon, lung and kidney epithelium, as well as in keratinocytes, and it seems to have a role in the defense of the organism, since it is activated by microorganisms or inflammatory mediators^43^. The finding that spores are able to bind to HaCaT cells suggests an activation of Nox, which will result in an increase of ROS levels and consequently in the nuclear translocation of Nrf-2. Accordingly, in our experimental system, no difference in p38 phosphorylation level was observed between control cells and cells incubated with spores after 75 min incubation (Fig. 3), whereas a small, although significant, increase was observed after 30 min incubation (Fig. 4). This result is in line with the hypothesis that the interaction between spores and cells gives rise to a transient, not toxic, activation of the oxidative stress pathway. The activation of the antioxidant system, through the up-regulation of Nrf-2, is critical for the protection of skin cells from oxidative stress-induced damage. Thus, our findings suggest that spores can be an effective component in the treatment of skin damage, photo-aging, and skin cancers.

## Methods

### *Bacillus subtilis* strains used and preparation of spores

*B. subtilis* wild type strain PY79 was used^44^. Sporulation was induced by exhaustion by growing cells in DSM (Difco Sporulation Medium) as described by Nicholson and Setlow^34^. After 30 h incubation at 37 °C, spores were collected, washed four times, incubated overnight in water at 4 °C to lyse residual sporangial cells and purified on a step gradient of 20% to 50% of Gastrografin^45^.

### Cell culture and cell survival assay

Human normal keratinocytes (HaCaT) and epithelial colorectal adenocarcinoma cells (LoVo) were obtained from ATCC. Both cell lines were cultured in Dulbecco’s Modified Eagle’s Medium (Sigma-Aldrich), supplemented with 10% fetal bovine serum (HyClone), 2 mM L-glutamine and antibiotics, all from Sigma-Aldrich, in a 5% CO_2_ humidified atmosphere at 37 °C. For sub-culturing cells, the culture medium was removed and cells were rinsed with PBS, detached with trypsinEDTA and diluted in fresh complete growth medium.

Cells were seeded in 96-well plates (100 μL/well) at a density of 2 × 10^3^/well (HaCaT cells). For dose-dependent survival assays, 24 h after seeding, increasing amount of spore (from 1:1 to 1:50) were added to the cells for 24–72 h. At the end of incubation, cells were detached by trypsin, centrifuged at 1000 g for 5 min at r.t. and the cell pellet was resuspended in 0.4% trypan blue buffer (Sigma-Aldrich) and counted in the hemocytometric chamber (Burker chamber, Sigma-Aldrich).

### Oxidative stress

To analyze oxidative stress, cells were plated at a density of 4 × 10^4^ cells/cm^2^ (LoVo cells) and 2 × 10^4^ cells/cm^2^ (HaCaT cells). 24 h after seeding, cells were incubated for 30 min in the presence or absence of PY79 spores (1:50, cells:spore), and then incubated in the presence of 300 µM SA for 45 min at 37 °C (treatment before injury). In a second group of experiments, cells were stressed with 300 µM SA for 45 min at 37 °C and then cell medium was changed to remove SA and cells were incubated for 30 min in the presence or absence of spores (treatment after injury). In case of UVA treatment, 24 h after seeding, cells were incubated for 30 min in the presence or absence of spores and then the medium was removed and cells were washed twice with PBS. After washing, cells were covered with a thin layer of PBS and irradiated for 2 min with UVA light (20 J/cm^2^). Subsequently to oxidative stress, cells were washed again with PBS and re-incubated with spores at 37 °C for 90 min.

### Measurement of intracellular ROS levels

To estimate ROS production, the protocol described in^46^ was followed. Briefly, at the end of incubation cells were incubated with a cells permeable probe, 2′,7′-dichlorodihydrofluorescein diacetate (H_2_-DCFDA, Sigma-Aldrich). The non fluorescent H_2_DCFDA becomes fluorescent product, 2′,7′-dichlorofluorescein (DCF), in the presence of different species of ROS. Fluorescence intensity was measured by a Perkin-Elmer LS50 spectrofluorimeter (525 nm emission wavelength, 488 nm excitation wavelength, 300 nm/min scanning speed, 5 slit width for both excitation and emission). ROS production was expressed as percentage of DCF fluorescence intensity of the sample under test, with respect to the untreated sample.

### Measurement of intracellular total GSH levels

Total activation of the GSH synthetic pathway was monitored by measuring GSH concentrations in the cells. Intracellular GSH levels were estimated as procedure previously was described^25^. Briefly, at the end of incubation, cells were detached by trypsin, lysed and protein concentration was determined by the Bradford assay. Then, 50 µg of proteins were incubated with 3 mM EDTA, 144 µM 5,5′-dithiobis-2-nitrobenzoic acid (DTNB) in 30 mM TrisHCl pH 8.2, centrifuged at 14,000 *g* for 5 min at 4 °C and the absorbance of the supernatant was measured at 412 nm by using a multiplate reader (Biorad). GSH levels were expressed as the percentage of TNB absorbance in the sample under test with respect to the untreated sample.

### Measurement of lipid peroxidation

The thiobarbituric acid reactive substances (TBARS) assay quantifies a by-product of lipid peroxidation, the malondialdehyde, that reacts with thiobarbituric acid (TBA) forming an abduct (MDA-TBA).

We used the protocol described by Del Giudice *et al*.^47^. Briefly, at the end of incubation, cells were kept for 90 min at 37 °C, then detached and suspended (5 × 10^4^ cells) in 0.67% thiobarbituric acid (TBA) and 20% trichloroacetic acid (1:1 v/v). After heating and centrifugation at 3000 g for 5 min at 4 °C, samples were read at 532 nm. Lipid peroxidation levels were expressed as the percentage of the absorbance at 532 nm of the sample under test, with respect to untreated cells (100%).

### Western blot analyses

HaCaT cells were plated at a density of 2 × 10^4^ cells/cm^2^ in complete medium for 24 h and then treated as described above (paragraph 4.3); 25 µM quercetin (Sigma-Aldrich) was used on HaCaT cells for 15 or 30 min. After treatment, total cell lysate was obtained by resuspend each cell pellet in 50 µL of lysis buffer (100 mM Tris-HCl, 300 mM NaCl and 0.5% NP40 at pH 7.4, with addiction of inhibitors of proteases and phosphatases). Nuclear pellet was obtained after extracting cytosolic proteins with PBS buffer containing 0.1% triton and proteases inhibitors. Nuclear lysate was obtained by resuspending the pellet in RIPA buffer (150 mM NaCl, 1% NP-40, 0.1% SDS, proteases inhibitors in 50 mM Tris-HCl pH8.0). Lysates (100 µg of proteins) were then analyzed by Western blotting performed as previously described^48^. Phosphorylation levels of p38, MAPKAPK-2, HSP-27 or total Nrf-2 were detected by using specific antibodies purchased from Cell Signal Technology (Danvers, MA, USA). HO-1 antibody was from Bethyl (Montgomery, TX, USA). To normalize protein intensity levels, a specific antibody against anti-GAPDH or anti B-23 (ThermoFisher, Rockford, IL, USA) were used for cytosolic and nuclear extracts, respectively. The chemiluminescence detection system (SuperSignal^®^ West Pico) was from Thermo Fisher.

### Catalase assay

Quantitative determination of catalase activity of spores and of cells after spore incubation was measured by the loss of absorbance at 240 nm as previously described by Beers and Sizer^49^. Briefly, spores (5 × 10^8^ or 1 × 10^9^) or cell lysate (50 µg) were incubated for 30 min at room temperature in 1 mL of hydrogen peroxide solution [50 mM Potassium Phosphate Buffer, pH 7.0, 0.036% (w/w) H_2_O_2_]. Then, samples were centrifuged for 1 min at 13000 g to remove the spores and the hydrogen peroxide concentration in solution was determined by measuring the absorbance at 240 nm. The percentage of peroxide removed was calculated as following:

$$ \% {{\rm{H}}}_{2}{{\rm{O}}}_{2}{\rm{rem}}=1-{{\rm{OD}}}_{240{\rm{nm}}}{\rm{sample}}/{{\rm{OD}}}_{240{\rm{nm}}}{\rm{standard}}.$$

Standard is referred to 1 ml of hydrogen peroxide solution.

### Spore adhesion assays

For adhesion assays, HaCaT cells were plated at a density of 3 × 10^4^ cells/cm^2^ in complete medium in a 24-well with cover glass for 24 h and then treated with spores previously bound with a red fluorescent protein^35^ for 30 min (1:50, cells:spores). At the end of incubation, cells were washed 3 times with 500 µL PBS to remove non-adherent bacteria and then fixed in 4% paraformaldehyde in PBS for 15 min. To verify the specificity of the binding between cells and spores, in a parallel experiment cells were incubated with 0.6 M NaCl in PBS for 10 min^50^ and then washed and fixed as described above. After fixing, cells were washed 3 times with 500 µL PBS and then observed with an Olympus BX51 fluorescence microscope. Images were captured using an Olympus DP70 digital camera equipped with Olympus U-CA Magnification Changer (100×) and processed with Image Analysis Software (Olympus) for minor adjustments of brightness, contrast and color balance and for creation of merged images.

To estimate the percentage of spores bound to cells, the same experiment described above was performed, but instead of fixing cells, cells were detached in trypsin and resuspended in PBS (2 × 10^4^ cells/mL). Fluorescence intensity of the cell suspension was measured by a Perkin-Elmer LS50 spectrofluorimeter (540 nm excitation wavelength, 625 nm emission wavelength, 300 nm/min scanning speed, 5 slit width for both excitation and emission). Rhodamine fluorescence was expressed as fluorescence intensity of the sample under test, and the fluorescence of untreated cells was subtracted from PY79- and PY79 + NaCl- treated cells. Alternatively, to determine the amount of spores bound to the cells, the procedure described by Batista and colleagues was followed^51^. Briefly, at the end of the experiment, cells were detached by trypsin, lysed and heated at 65 °C for 30 min and then plated on LB-agar plates. After incubation at 37 °C over-night, CFUs were counted.

### Statistical analyses

In all the experiments samples were analyzed in triplicate. The results are presented as mean of results obtained after at least three independent experiments (mean ± SD) and compared by one-way ANOVA following Tukey’s multiple comparison test using Graphpad Prism for windows, Version 6.01.

## Electronic supplementary material

Supplementary material

## References

[1] Chen C, Jiang X, Zhao W, Zhang ZZ (2013). Dual role of resveratrol in modulation of genotoxicity induced by sodium arsenite via oxidative stress and apoptosis. Food Chem. Toxicol..

[2] Rossman TG (2003). Mechanism of arsenic carcinogenesis: An integrated approach. Mutat. Res. - Fundam. Mol. Mech. Mutagen..

[3] Hughes MF, Beck BD, Chen Y, Lewis AS, Thomas DJ (2011). Arsenic exposure and toxicology: A historical perspective. Toxicol. Sci..

[4] Cui X, Kobayashi Y, Akashi M, Okayasu R (2008). Metabolism and the Paradoxical Effects of Arsenic: Carcinogenesis and Anticancer. Curr. Med. Chem..

[5] Smith AH (1992). Cancer risks from arsenic in drinking water. Environ. Health Perspect..

[6] Rahman MM, Ng JC, Naidu R (2009). Chronic exposure of arsenic via drinking water and its adverse health impacts on humans. Environ. Geochem. Health.

[7] Brown KG, Ross GL (2002). Arsenic, Drinking Water, and Health: A Position Paper of the American Council on Science and Health. Regul. Toxicol. Pharmacol..

[8] Ruiz-Ramos R, Lopez-Carrillo L, Rios-Perez AD, De Vizcaya-Ruíz A, Cebrian ME (2009). Sodium arsenite induces ROS generation, DNA oxidative damage, HO-1 and c-Myc proteins, NF-κB activation and cell proliferation in human breast cancer MCF-7 cells. Mutat. Res. - Genet. Toxicol. Environ. Mutagen..

[9] Zhang Z (2011). Reactive oxygen species mediate arsenic induced cell transformation and tumorigenesis through Wnt/β-catenin pathway in human colorectal adenocarcinoma DLD1 cells. Toxicol. Appl. Pharmacol..

[10] Imlay JA (2003). Pathways of oxidative damage. Annu. Rev. Microbiol..

[11] Hei TK, Filipic M (2004). Role of oxidative damage in the genotoxicity of arsenic. Free Radic. Biol. Med..

[12] Wang TS, Kuo CF, Jan KY, Huang H (1996). Arsenite induces apoptosis in Chinese hamster ovary cells by generation of reactive oxygen species. J. Cell. Physiol..

[13] Cutting SM, Hong Ha, Baccigalupi L, Ricca E (2009). 18. Oral vaccine delivery by recombinant spore probiotics. Int. Rev. Immunol..

[14] Isticato R (2013). Non-recombinant display of the B subunit of the heat labile toxin of Escherichia coli on wild type and mutant spores of Bacillus subtilis. Microb. Cell Fact..

[15] Green, D. H. *et al*. Characterization of Two Bacillus Probiotics Characterization of Two Bacillus Probiotics. **65**, 8–12 (1999).

[16] Rhee K-J, Sethupathi P, Driks A, Lanning DK, Knight KL (2004). Role of commensal bacteria in development of gut-associated lymphoid tissues and preimmune antibody repertoire. J. Immunol..

[17] Mazza P (1994). The use of Bacillus subtilis as an antidiarrhoeal microorganism. Boll. Chim. Farm..

[18] Ara K (2006). Foot odor due to microbial metabolism and its control. Can. J. Microbiol..

[19] Earl AM, Losick R, Kolter R (2008). Ecology and genomics of Bacillus subtilis. Trends Microbiol..

[20] Stein T (2005). Bacillus subtilis antibiotics: structures, syntheses and specific functions. Mol. Microbiol..

[21] Liu W-T (2010). Imaging mass spectrometry of intraspecies metabolic exchange revealed the cannibalistic factors of Bacillus subtilis. Proc. Natl. Acad. Sci. USA.

[22] Alebouyeh M (2009). Characterization of the interaction of undomesticatedBacillus subtilis spores with Caco-2 cell line. Ann. Microbiol..

[23] Simon H-U, Haj-Yehia A, Levi-Schaffer F (2000). Role of reactive oxygen species (ROS) in apoptosis induction. Apoptosis.

[24] Anderson ME (1998). Glutathione: An overview of biosynthesis and modulation. Chem. Biol. Interact..

[25] Petruk G (2016). An ascorbic acid-enriched tomato genotype to fight UVA-induced oxidative stress in normal human keratinocytes. J. Photochem. Photobiol. B Biol..

[26] Liu X (2014). p38 and Extracellular Signal-Regulated Kinases Activations have Opposite Effects on Primary-Cultured Rat Cerebellar Granule Neurons Exposed to Sodium Arsenite. J. Biochem. Mol. Toxicol..

[27] Duyndam MCA, Hulscher STM, van der Wall E, Pinedo HM, Boven E (2003). Evidence for a role of p38 kinase in hypoxia-inducible factor 1-independent induction of vascular endothelial growth factor expression by sodium arsenite. J. Biol. Chem..

[28] Ma Q (2013). Role of nrf2 in oxidative stress and toxicity. Annu. Rev. Pharmacol. Toxicol..

[29] Jones RM (2013). Symbiotic lactobacilli stimulate gut epithelial proliferation via Nox-mediated generation of reactive oxygen species. EMBO J..

[30] Jones RM (2015). Lactobacilli Modulate Epithelial Cytoprotection through the Nrf2 Pathway. Cell Rep..

[31] Hseu YC (2012). Ellagic acid protects human keratinocyte (HaCaT) cells against UVA-induced oxidative stress and apoptosis through the upregulation of the HO-1 and Nrf-2 antioxidant genes. Food Chem. Toxicol..

[32] Chow J-M, Shen S-C, Huan SK, Lin H-Y, Chen Y-C (2005). Quercetin, but not rutin and quercitrin, prevention of H2O2-induced apoptosis via anti-oxidant activity and heme oxygenase 1 gene expression in macrophages. Biochem. Pharmacol..

[33] Kang C-H, Choi YH, Moon S-K, Kim W-J, Kim G-Y (2013). Quercetin inhibits lipopolysaccharide-induced nitric oxide production in BV2 microglial cells by suppressing the NF-κB pathway and activating the Nrf2-dependent HO-1 pathway. Int. Immunopharmacol..

[34] Colin, R. Harwood, S. M. C. *Molecular biological methods for Bacillus*. (1990).

[35] Donadio G, Lanzilli M, Sirec T, Ricca E, Isticato R (2016). Localization of a red fluorescence protein adsorbed on wild type and mutant spores of Bacillus subtilis. Microb. Cell Fact..

[36] Faussner A (2012). Binding characteristics of [3H]-JSM10292: a new cell membrane-permeant non-peptide bradykinin B2 receptor antagonist. Br. J. Pharmacol..

[37] Singh N, Kumar D, Sahu AP (2007). Arsenic in the environment: effects on human health and possible prevention. J. Environ. Biol..

[38] Alam A (2014). Redox signaling regulates commensal-mediated mucosal homeostasis and restitution and requires formyl peptide receptor 1. Mucosal Immunol..

[39] Wentworth CC, Jones RM, Kwon YM, Nusrat A, Neish AS (2010). Commensal-Epithelial Signaling Mediated via Formyl Peptide Receptors. Am. J. Pathol..

[40] Wentworth CC, Alam A, Jones RM, Nusrat A, Neish AS (2011). Enteric commensal bacteria induce extracellular signal-regulated kinase pathway signaling via formyl peptide receptor-dependent redox modulation of dual specific phosphatase 3. J. Biol. Chem..

[41] Swanson PA (2011). Enteric commensal bacteria potentiate epithelial restitution via reactive oxygen species-mediated inactivation of focal adhesion kinase phosphatases. Proc. Natl. Acad. Sci. USA.

[42] Kumar A (2007). Commensal bacteria modulate cullin-dependent signaling via generation of reactive oxygen species. EMBO J..

[43] Lambeth JD (2004). NOX enzymes and the biology of reactive oxygen. Nat. Rev. Immunol..

[44] Youngman P, Perkins JB, Losick R (1984). A novel method for the rapid cloning in Escherichia coli of Bacillus subtilis chromosomal DNA adjacent to Tn917 insertions. Mol. Gen. Genet..

[45] Henriques AO, Melsen LR, Moran CP (1998). Involvement of superoxide dismutase in spore coat assembly in Bacillus subtilis. J. Bacteriol..

[46] Del Giudice R (2017). Carotenoids in fresh and processed tomato (Solanum lycopersicum) fruits protect cells from oxidative stress injury. J. Sci. Food Agric..

[47] Del Giudice R (2015). Antioxidant bioactive compounds in tomato fruits at different ripening stages and their effects on normal and cancer cells. J. Funct. Foods.

[48] Guglielmi, F. *et al*. Enzymatically active fibrils generated by the self-assembly of the ApoA-I fibrillogenic domain functionalized with a catalytic moiety. *Biomaterials* **30** (2009). 10.1016/j.biomaterials.2008.10.036 19027944

[49] Beers R, Sizer I (1952). A spectrophotometric method for measuring the breakdown of hydrogen peroxide by catalase. J. Biol. Chem..

[50] Arciello A (2011). Insights into the fate of the N-terminal amyloidogenic polypeptide of ApoA-I in cultured target cells. J. Cell. Mol. Med..

[51] Tavares Batista M (2014). Gut adhesive Bacillus subtilis spores as a platform for mucosal delivery of antigens. Infect. Immun..

