# Supplementary material for: Alternative use of Bacillus subtilis spores: protection against environmental oxidative stress in human normal keratinocytes
Source: Sci Rep. 2018 Jan 29;8:1745. doi: 10.1038/s41598-018-20153-2 (PMC5788939; doi:10.1038/s41598-018-20153-2)
Supplement: Supplementary file 1 — Supplementary material [file 41598_2018_20153_MOESM1_ESM.pdf]

Alternative use of *Bacillus subtilis* spores: protection against environmental oxidative stress in human normal keratinocytes

Ganna Petruk[a], Giuliana Donadio[b], Mariamichela Lanzilli[b], Rachele Isticato[b]*, Daria Maria Monti [a,c]*

[a] Department of Chemical Sciences, University of Naples Federico II, Complesso Universitario Monte Sant'Angelo, via Cinthia 4, 80126, Naples, Italy

[b]Department of Biology, University of Naples Federico II, Complesso Universitario Monte Sant'Angelo, via Cinthia 4, 80126, Naples, Italy

[c]Istituto Nazionale di Biostrutture e Biosistemi (INBB), Rome, Italy

***** Corresponding authors. R. Isticato, E-mail: rachele.isticato@unina.it; D.M. Monti, E-mail: mdmonti@unina.it.

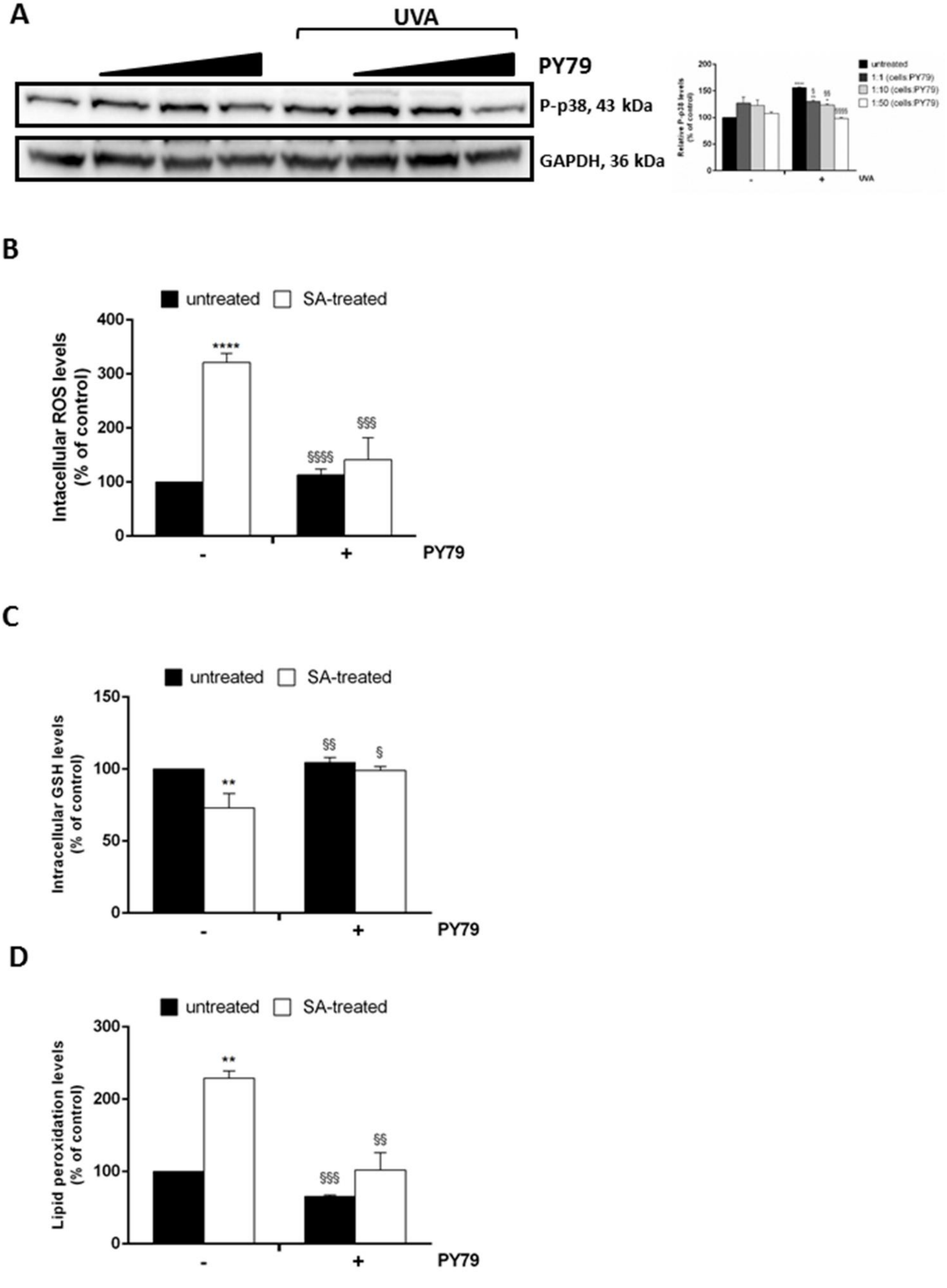
A
UVA
PY79
P-p38, 43 kDa
GAPDH, 36 kDa
Relative P-p38 levels (% of control)
untreated
1:1 (cells:PY79)
1:10 (cells:PY79)
1:50 (cells:PY79)
UVA
B
untreated
SA-treated
Intracellular ROS levels (% of control)
PY79
C
untreated
SA-treated
Intracellular GSH levels (% of control)
PY79
D
untreated
SA-treated
Lipid peroxidation levels (% of control)
PY79

Figure S1: Analysis of oxidative stress in HaCaT cells exposed to UVA treatment and LoVo cells stressed by SA in the presence of PY79 spores. **A**, HaCaT cells were pre-incubated in the presence of increasing amount of PY79 (from 1:1 to 1:50, cells:spores) for 30 min and then irradiated by UVA (20 J/cm^2). Western blots show the phosphorylation level of p38, with the relative densitometric analysis in the absence (-) or in the presence (+) of UVA stress. GAPDH was used as internal standard. * indicates p<0.05, ** indicates p<0.01, **** indicates p<0.0001, with respect to control cells; § indicates p<0.05, §§ indicates p<0.01, §§§§ indicates p<0.0001, with respect to UVA-treated cells. **B-D**, LoVo cells incubated with PY79 (1:50, cells:spores) for 30 min before treatment with 300 µM SA for 45 min. **B**, DCFDA assay; **C**, DTNB assay; **D**, TBARS assay. Values are expressed as fold increase with respect to control cells. Data shown are the means ± S.D. of three independent experiments. ** indicates p<0.005, **** indicates p<0.0001, with respect to control cells; § indicates p<0.05, §§ indicates p<0.01, §§§ indicates p<0.001, §§§§ indicates p<0.0001, with respect to SA-treated cells.

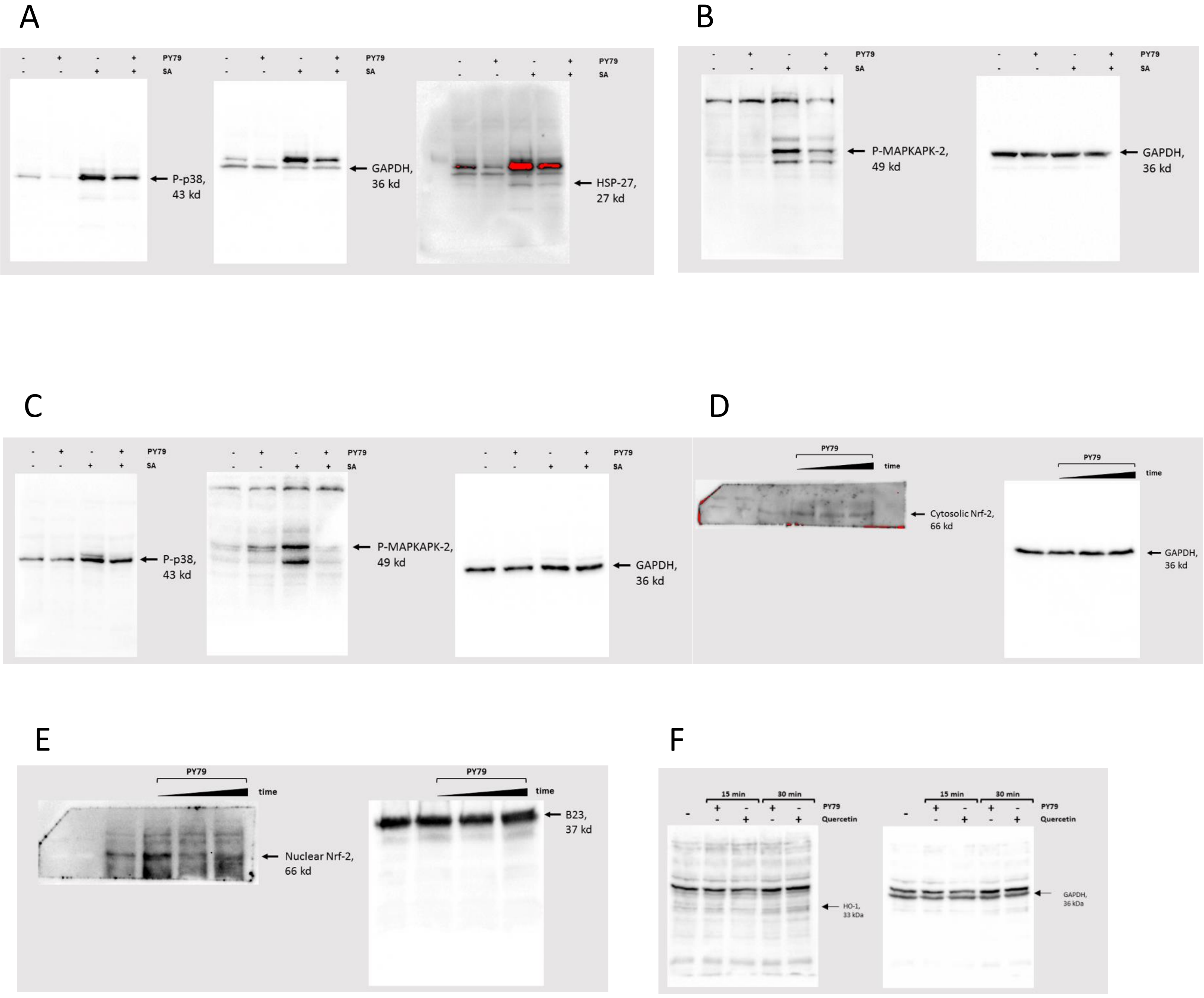

Figure S2: Uncropped blots corresponding to main Figures 3-5. All SDS-PAGE were run under the same experimental conditions. Following transfer, membranes were either subsequently incubated with antibodies or cut into strips to minimize the required amount of antibody. Arrows indicate were blots were cropped.

(A-B) Uncropped blots for images shown in Fig. 3.

(C) Uncropped blots for images shown in Fig. 4.

(D-F) Uncropped blots for images shown in Fig. 5.
